# A robust qualitative transcriptional signature for the correct pathological diagnosis of gastric cancer

**DOI:** 10.1186/s12967-019-1816-4

**Published:** 2019-02-28

**Authors:** Haidan Yan, Meifeng Li, Longlong Cao, Haifeng Chen, Hungming Lai, Qingzhou Guan, Huxing Chen, Wenbin Zhou, Baotong Zheng, Zheng Guo, Chaohui Zheng

**Affiliations:** 1 Department of Bioinformatics, Key Laboratory of Ministry of Education for Gastrointestinal Cancer, School of Basic Medical Sciences, Fujian Medical University, Fuzhou, 350122 China; 2 Department of Gastric Surgery, Fujian Medical University Union Hospital, No. 29 Xinquan Road, Fuzhou, 350001 China; 3 Department of General Surgery, Fuzhou Second Hospital Affiliated To Xiamen University, Xiamen, 350007 China; 4 Department of Systems Biology, College of Bioinformatics Science and Technology, Harbin Medical University, Harbin, 150086 China

**Keywords:** Gastric cancer, Gastritis, Gastroscopy biopsy, Diagnosis, Signature

## Abstract

**Background:**

Currently, pathological examination of gastroscopy biopsy specimens is the gold standard for gastric cancer (GC) diagnosis. However, it has a false-negative rate of 10–20% due to inaccurate sampling locations and/or insufficient sampling amount. A signature should be developed to aid the early diagnosis of GC using biopsy specimens even when they are sampled from inaccurate locations.

**Methods:**

We extracted a robust qualitative transcriptional signature, based on the within-sample relative expression orderings (REOs) of gene pairs, to discriminate both GC tissues and adjacent-normal tissues from non-GC gastritis, intestinal metaplasia and normal gastric tissues.

**Results:**

A signature consisting of two gene pairs for GC diagnosis was identified and validated in data of both biopsy specimens and surgical resection specimens pooled from publicly available datasets measured by different laboratories with different platforms. For gastroscopy biopsy specimens, 96.20% of 79 non-GC tissues were correctly identified as non-GC, and 96.84% of 158 GC tissues and six of seven adjacent-normal tissues were correctly identified as GC. For surgical resection specimens, 98.37% of 2560 GC tissues and 97.28% of 221 adjacent-normal tissues were correctly identified as GC. Especially, 97.67% of the 257 GC patients at stage I were exactly diagnosed as GC. We additionally measured 21 GC tissues from seven different GC patients, each with three specimens sampled from three tumor locations with different proportions of the tumor epithelial cell. All these GC tissues were correctly identified as GC, even when the proportion of the tumor epithelial cell was as low as 14%.

**Conclusions:**

The qualitative transcriptional signature can distinguish both GC and adjacent-normal tissues from normal, gastritis and intestinal metaplasia tissues of non-GC patients even using inaccurately sampled biopsy specimens, which can be applied robustly at the individual level to aid the early GC diagnosis.

**Electronic supplementary material:**

The online version of this article (10.1186/s12967-019-1816-4) contains supplementary material, which is available to authorized users.

## Background

Gastric cancer (GC) is one of the most frequent malignant tumors with a high mortality rate [1–3]. GC patients at early stage could benefit from surgical resection [4, 5]. However, only about 10–20% of GC patients are diagnosed at early stage [6, 7]. Currently, pathological examination based on gastroscopic biopsy tissue is still the most effective approach for confirming GC [8, 9]. However, the result of pathological examination for gastroscopic biopsy tissue depends on the skills and experiences of the endoscopists and pathologists [10–12]. The false-negative rate of GC diagnosis has been reported to be 10–20% [13–18]. Among the false-negative samples, 85.2% are at the early stage [19], and 71.4% are wrongly diagnosed as gastritis, ulcer or “suspicious lesion” [16]. Most of the false-negative samples (73%) are caused by inaccurate sampling locations and the remainder (27%) could be attributed to pathologist errors [16].

Therefore, it is vitally important to develop an objective molecular signature to complement the existing subjective diagnostic technique of histology, which could aid the pathologists to identify early GC even when the sampling location of gastroscopic biopsy tissue is inaccurate. It’s possible because the GC adjacent-normal tissues might also gain some similar molecular characteristics of GC [20, 21]. However, most of the reported diagnostic signatures are identified using GC adjacent-normal tissues as the normal samples [22–24], which will make false-negative diagnosis when the location of gastroscopic biopsy tissue is inaccurate [13]. Another critical limitation of previously reported diagnostic signatures is that they are based on risk scores summarized from quantitative gene expression measurements of the signature genes [22, 23, 25], which are highly sensitive to measurement batch effects and lab differences and thus cannot be robustly applied to independent samples [26–28] even with data normalization [29]. Fortunately, it has been reported that the within-sample relative expression orderings (REOs) of genes are robust against experimental batch effects [30, 31]. Besides, we have shown that the within-sample REOs are robust even when the tumor tissues sampled from different tumor locations contain different proportions of the tumor epithelial cell [32] and partial RNA degradation during specimen preparation and storage [33], and the RNA amplification bias exists for minimum specimens. Notably, Zheng et al. have identified the within-sample REO of one pair of microRNA (hsa-miR-196a and hsa-miR-148a) as a qualitative GC diagnosis signature using GC and normal gastric mucosa samples [34]. However, the performance of this signature to identify gastritis, intestinal metaplasia and cancer adjacent-normal samples was not evaluated [34].

In this study, we aim at identifying a signature that can discriminate GC tissues, including the inaccurately sampled GC adjacent-normal tissues, from non-GC tissues including gastritis, intestinal metaplasia and normal gastric tissues. A signature consisting of two gene pairs was identified in the training data and validated in multiple datasets measured by different laboratories with different platforms, even when the proportion of the tumor epithelial cell was as low as 14%.

## Materials and methods

### Samples and data measurement

We measured 21 GC specimens from seven GC patients. For each patient, three specimens were sampled from three different tumor locations. The proportion (about 14%–93%) of the tumor epithelial cell was measured by pathological section analysis (see Table 1). The baseline characteristics of the seven GC patients were shown in Additional file 1: Table S1. All cancer specimens were collected from the operating room immediately after surgical resection and were fresh frozen for subsequent RNA extraction. This study was approved by the institutional review boards of all participating institutions, and written consent forms were obtained from all participants.

**Table 1:** The proportions of the tumor epithelial cell for GC tissues of each patient sampled from three different locations

Patient	Proportion 1 (%)	Proportion 2 (%)	Proportion 3 (%)
GC 1	23	79	53
GC 2	53	28	89
GC 3	27	73	93
GC 4	35	67	89
GC 5	88	37	14
GC 6	88	33	57
GC 7	15	74	47

Total RNA was isolated from fresh frozen GC tissues using Trizol reagent (Invitrogen) according to the manufacture’s protocol. The quality of RNA was assessed using Agilent 2200 TapeStation (Agilent technologies, US) to ensure high quality (RNA integrity number > 6). Then, 1–2 μg of total RNA was used for mRNA capture using NEBNextPolyA mRNA Magnetic Isolation Module and stranded RNA-seq libraries were constructed using a NEBNext Ultra Directional RNA Library Prep Kit. The 2 × 150 paired-end sequencing was performed on an Illumina HiSeqXten (Illumina, US). The resulting raw RNA-seq files (.fastq) were preprocessed using Trimmomatic [35], and reads were aligned to the reference genome (GRCh37) using hisat2 [36]. Finally, the reads per kilobase per million mapped reads (RPKM) values of genes were computed to represent the expression levels of genes using StringTie [37]. The data has been submitted to Gene Expression Omnibus (GEO, GSE116782).

### Public data and preprocessing

Gene expression profiles of gastric tissues measured by the Affymetrix, Illumina or RNA-seq platform were collected from the GEO and The Cancer Genome Atlas (TCGA) data portal (http://tcga-data.nci.nih.gov/tcga/), as described in Table 2.

**Table 2:** The publicly available datasets used in the study

Dataset	Platform	Normal	GI	GC	GC_adjacent
Training					
GSE54129	Afftmetrix GPL570	21^a^	–	111	–
GSE54043	Afftmetrix GPL570	5^a^	5^a^	–	–
GSE42252	Afftmetrix GPL570	–	–	5	–
GSE38749	Afftmetrix GPL570	–	–	15	–
GSE51725	Afftmetrix GPL570	–	–	8	–
GSE79973	Afftmetrix GPL570	–	–	10	–
GSE57303	Afftmetrix GPL570	–	–	70	–
GSE13911	Afftmetrix GPL570	–	–	38	–
GSE27411	Illumina GPL6255	–	18^a^	–	–
GSE28541	Illumina GPL13376	–	–	40	–
GSE29998	Illumina GPL6947	–	–	50	–
Total		26	23	347	–
Validation					
GSE5081	Afftmetrix GPL570	–	32^a^	–	–
GSE52138	Afftmetrix GPL96	–	–	13^a^	7^a^
GSE14210	Afftmetrix GPL571	–	–	145^a^	–
GSE106656	Afftmetrix GPL6244	–	21^a^	–	–
GSE34619	Afftmetrix GPL6244	10^a^	–	–	–
GSE29272	Afftmetrix GPL96	–	–	134	134
GSE34942	Afftmetrix GPL570	–	–	56	–
GSE22377	Afftmetrix GPL570	–	–	43	–
GSE19826	Afftmetrix GPL570	–	–	12	12
GSE35809	Afftmetrix GPL570	–	–	70	–
GSE51105	Afftmetrix GPL570	–	–	94	–
GSE15459	Afftmetrix GPL570	–	–	200	–
GSE62254	Afftmetrix GPL570	–	–	300	–
GSE13861	Illumina GPL6884	–	–	65	19
GSE38024	Illumina GPL10558	–	–	48	–
GSE26899	Illumina GPL6947	–	–	96	12
GSE26253	Illumina GPL8432	–	–	432	–
GSE84437	Illumina GPL6947	–	–	433	–
GSE26942	Illumina GPL6947	–	–	202	12
GSE60662	Agilent GPL13497	4^a^	12^a^	–	–
TCGA	RNA-seq	–	–	375	32
Total		14	65	2718	228

GI represent gastritis, gastritis adjacent-normal or intestinal metaplasia tissues. GC_adjacent represent the GC adjacent-normal tissues

^a^Denotes the samples were collected by gastroscopic biopsy

For the gene expression profiles measured by the Affymetrix platform, the raw data (.CEL files) was downloaded and preprocessed using the Robust Multi-array Average algorithm for background adjustment without quantile normalization [38]. For the gene expression profiles measured by the Illumina platform, the processed data was directly downloaded and used for the following analysis. For the gene expression profiles from TCGA detected by RNA-seq, the level 3 data was directly downloaded for our analysis.

For the array-based data, every probe ID was mapped to Entrez gene ID using the corresponding platform file. If multiple probes were mapped to a gene, the expression level of this gene was summarized as the arithmetic mean of the values of these probes.

### Developing the diagnostic signature

The gene expression profiles of GC, normal and gastritis tissues in the training data were used to identify REO-based diagnostic signature (Table 2). First, we defined the stable REOs of gene pairs in a type of gastric tissues. The REO of a gene pair (*i*, *j*) is denoted as *Gi *> *Gj* or Gi < *Gj* if the gene *i* has a higher or lower expression level than the gene *j* within a sample. The REO of a gene pair is defined as stable if the same REO kept in at least 99% of the samples. Furthermore, a gene pair (*i*, *j*) is defined as reversal if the REO of the gene pair is stable in both of two types of gastric tissues, but with different REO patterns (*Gi* < *Gj* or *Gi *> *Gj* in one type of tissues but *Gi* > *Gj* or *Gi *< *Gj* in the other type of tissues). Here, the stable gene pairs with the same REO pattern between normal samples and gastritis samples were defined as stable gene pairs of non-GC tissues. We then selected the reversal gene pairs between GC and non-GC tissue samples. These reversal gene pairs were the candidate qualitative REO-based diagnostic signatures. The absolute rank difference for every reversal gene pair in each of the GC or non-GC samples is calculated as follow:

$$ R_{ij} = |R_{i} - R_{j} | $$

where *R*_*i*_ and *R*_*j*_ represent the ranks of gene *i* and *j* in a sample, respectively.

For a reversal gene pair (*i*, *j*), let *mean* [*R*_*ij*_(*non*)] and *mean* [*R*_*ij*_(*gc*)] denote the means of the absolute rank differences between gene *i* and gene *j* in non-GC tissue samples and GC tissue samples, respectively. Then, their geometric mean (*avgR*_*ij*_) is calculated to evaluate the reversal degree of the gene pair between GC and non-GC tissue samples.

$$ avgR_{ij} = \sqrt {mean[R_{ij} (non)] \times mean[R_{ij} (gc)]} $$

The larger the geometric mean for a reversal gene pair, the larger the reversal degree of the REO of the gene pair between GC and non-GC tissue samples. All reversal gene pairs were sorted in a descending order according to the geometric means.

Finally, we took the top *k* reversal gene pairs as a signature according to the reversal degrees of the identified reversal gene pairs, and a given sample was identified as GC tissue when at least a half of gene pairs in the signature exhibit the same REOs for GC; otherwise, it was identified as non-GC tissue. The signature achieved the highest classification accuracy in the training data was defined as GC diagnosis signature. All the analysis programs to develop the diagnostic signature were written using the R language (R 3.1.3). The program codes were shown in Additional file 2.

### Performance evaluation

The sensitivity, specificity, accuracy and the area under curve (AUC) of the receiver operating characteristic (ROC) curves were used to evaluate the performance of the signature. The sensitivity was defined as the proportion of correctly identified GC samples in all GC samples. The specificity was defined as the proportion of correctly identified non-GC samples in all non-GC samples including normal tissues, gastritis adjacent-normal tissues and gastritis tissues. The accuracy was defined as the proportion of correctly identified samples of all GC and non-GC samples. Here, the nonparametric Hanley-McNeil algorithm was used to calculate the AUC value [39, 40] and 95% confidence intervals (CI) for AUC was computed using an approximate normal distribution.

## Results

### Identifying the diagnostic gene pair signature

**Fig. 1:**
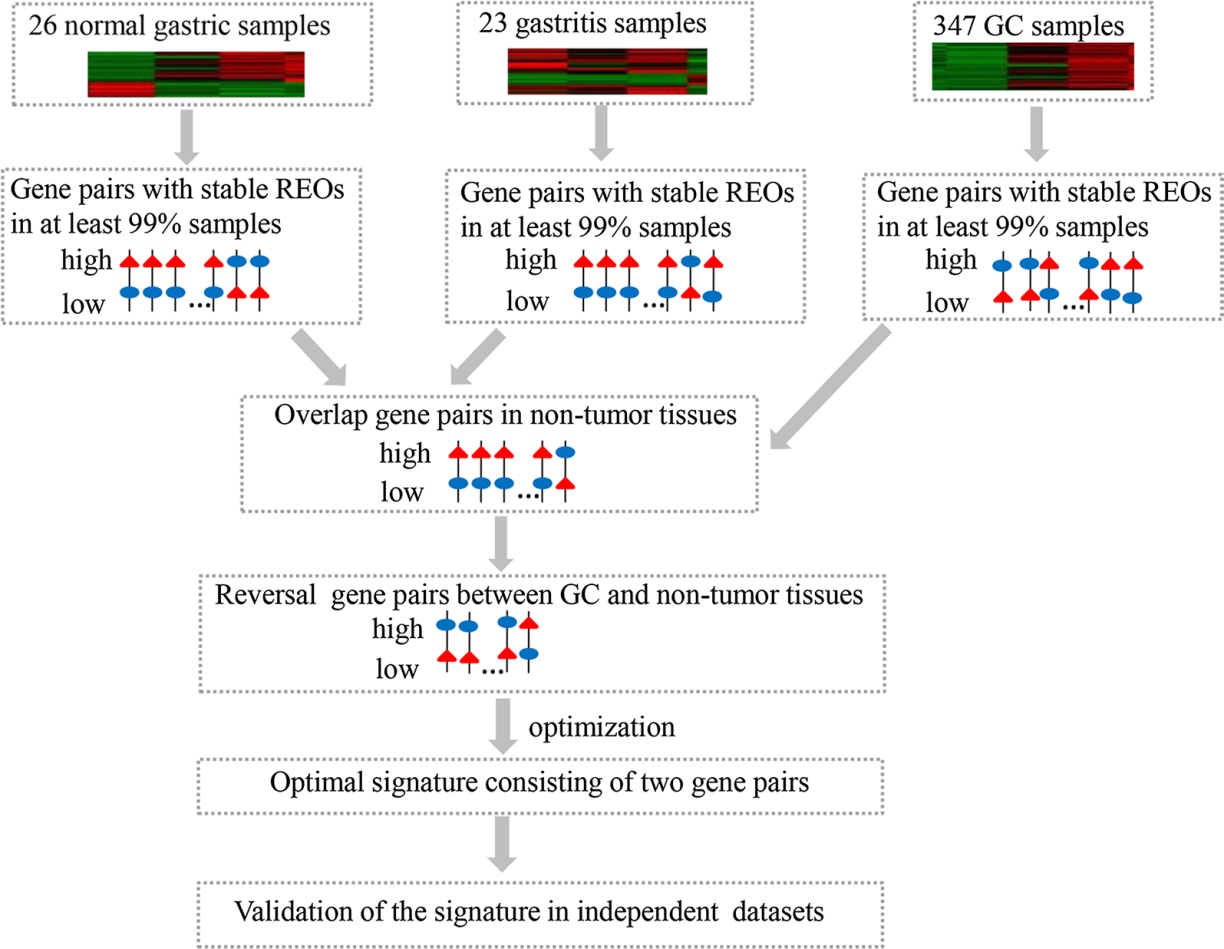
Outline of the processes for developing and validating the GC diagnosis signature

The flowchart for the identification and validation of the qualitative diagnostic signature is described in Fig. 1.

Firstly, we identified gene pairs with an identical REO in at least 99% of 26 gastric normal samples, 23 gastritis samples and 347 GC samples, respectively, using the training data integrated from 11 datasets measured by the Affymetrix or Illumina platform (see Table 1). We found 32,483,417 overlapped gene pairs with the same stable REOs between the gastric normal and gastritis samples, among which six gene pairs had stable but reversal REOs in the GC tissues (Additional files 3 and 4), which were potential GC diagnostic signatures.

**Fig. 2:**
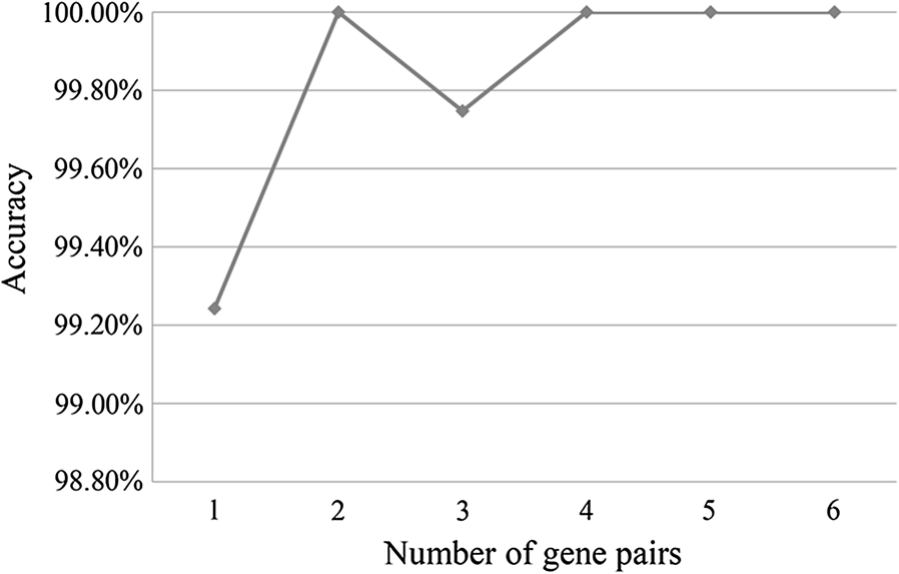
The classification accuracy of the top k gene pairs in the training data

We then evaluated the reversal degrees of the six gene pairs with reversal REOs between the GC and non-GC samples including normal and gastritis samples in the training data (see Methods). According to the reversal degrees of the six gene pairs, we took the top *k* (1, 2,…, 6) gene pairs as a signature and calculated its classification accuracy (Fig. 2). Finally, the top two gene pairs consisting of three genes, were defined as the diagnosis signature (Table 3). In the training data, all the 26 gastric normal and 23 gastritis tissues were correctly classified as non-GC samples, and all the 347 GC tissues were correctly classified as cancer samples. The AUC and the accuracy were 0.99 and 100%, respectively. The detailed classification accuracy of the signature in each of the training datasets was shown in Additional file 5: Table S3.

**Table 3:** The signature of gene pairs for GC diagnosis

Gene pairs	Gene A	Gene B
Pair 1	CYR61	MMP28
Pair 2	CYR61	ACOX1

The expression level of Gene A is higher than that of Gene B in GC patients

### Validating the signature

The gene expression profiles of gastric tissues sampled by gastroscopic biopsy or surgical resection were used to validate the performance of the qualitative signature.

**Fig. 3:**
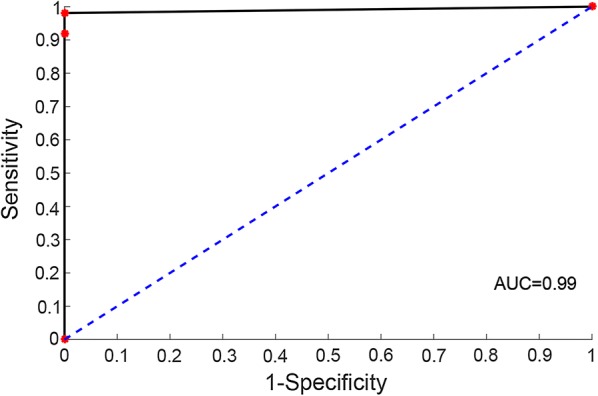
The receiver characteristic operating curves

Non-GC tissues, including normal, gastritis adjacent-normal, gastritis and intestinal metaplasia tissues, from non-GC patients were all sampled by gastroscopic biopsy. The result showed that 96.20% of the 79 non-GC tissues from GSE5081, GSE60662, GSE106656 and GSE34619 were correctly identified as non-GC (Table 4 and Additional file 6). For gastroscopic biopsy specimens, 96.84% of the 158 GC tissues from the GSE14210 and GSE52138 datasets and six of seven GC adjacent-normal tissues from the GSE52138 dataset were correctly identified as GC (Table 4 and Additional file 6). For surgical resection specimens, as described in Table 2, 98.37% of 2560 GC tissues and 97.28% of 221 samples were correctly identified as GC (Table 4). The surgical resection specimens were measured by multiple platforms including the Affymetrix, Illumina and RNA-seq platforms. For the Affymetrix and Illumina platforms used in training data, 99.77% of the 2185 GC tissues and all the 189 GC adjacent-normal tissues were correctly classified to GC tissues. Moreover, 95.73% of the 375 GC tissues and 81.25% of the 32 GC adjacent-normal tissues measured by RNA-seq were correctly classified to GC given that no RNA-seq data participated in training the signature. Especially, 97.67% of the 257 GC patients at stage I were correctly identified as GC. The accuracy and AUC of the validation data were 98.55% and 0.99 (95% CI = 0.95–1, Fig. 3).

**Table 4:** The performance of the signature in each of the validation datasets

Platforms	Dataset	Number (sensitivity) of GC tissues	Number (specificity) of non-GC tissues
Affymetrix	GSE5081^a^	–	32 (100.00%)
	GSE52138^a^	13 (92.31%)	–
	GSE14210^a^	145 (97.24%)	–
	GSE106656^a^	–	21 (90.48%)
	GSE34619^a^	–	10 (100.00%)
	GSE34942	56 (100.00%)	–
	GSE22377	43 (100.00%)	–
	GSE29272	134 (100.00%)	–
	GSE19826	12 (100.00%)	–
	GSE35809	70 (100.00%)	–
	GSE51105	94 (100.00%)	–
	GSE15459	200 (100.00%)	–
	GSE62254	300 (99.00%)	–
Illumina	GSE13861	65 (100.00%)	–
	GSE38024	48 (97.92%)	–
	GSE26899	96 (100.00%)	–
	GSE26253	432 (98.84%)	–
	GSE84437	433 (98.15%)	–
	GSE26942	202 (100.00%)	–
Agilent	GSE60662^a^	–	16 (93.75%)
RNA-seq	TCGA	375 (95.73%)	–
	Our-data	21 (100.00%)	–

^a^Denotes the samples collected by gastroscopic biopsy

To further validate the signature, using RNA-seq platform, we additionally measured gene expression profiles of 21 GC tissues from seven different GC patients, each with three specimens sampled from three tumor locations with different proportions of the tumor epithelial cell (see Table 1). All the 21 GC tissues were correctly classified to GC by our signature, even when the proportion of the tumor epithelial cell was as low as 14% (Table 4).

Together, the above results validated that the signature can accurately discriminate GC, including GC adjacent-normal tissues, from non-GC patients, even when the sampling location is inaccurate.

## Discussion

At present, the histological analysis of the gastroscopic biopsy specimen is affected by the sampling location and tissue amount [8]. In this study, a robust qualitative transcriptional signature, including two gene pairs consisting of three genes, was developed to aid the early diagnosis of GC using either gastroscopic biopsy or surgical resection specimens. The signature can accurately distinguish GC tissues from non-GC tissues including normal, gastritis and intestinal metaplasia tissues. As shown in this study, the signature can accurately classify GC tissues to GC when the proportion of the tumor epithelial cell was as low as 14%. Especially, it can identify most of GC adjacent-normal tissues as cancer, suggesting that the signature can identify GC even when the sampling location is inaccurate. Notably, all the non-GC tissues sampled by gastroscopic biopsy can be correctly identified as non-GC. However, the specimens sampled by gastroscopic biopsy for gastritis and intestinal metaplasia are limited, and it deserves further studies using large collections of non-GC specimens.

The amount of the gastroscopic biopsy specimens used in the study was about 1–8 µg total RNA [41–43] which was relatively large. In clinical practice, it is often difficult to obtain sufficient amount of biopsy specimens for gene expression profiling or other molecular measurements [11, 44]. Fortunately, we have shown that the REO-based signatures can be robustly applied to specimens with RNA amplification from as low as 150–250 pg total RNA of cancer cells [31]. Therefore, it is highly possible that the two gene pairs could be used to gastroscopic biopsy specimens with minimum sampling amounts. We compared the expression levels of the two genes in each of the signature gene pairs. The fold changes (FC) of the two genes in each of the signature gene pairs across different datasets for the GC, GC adjacent-normal and non-GC groups were quite different (Additional files 7 and 8). For the gene pair of CYR61 and MMP28, the median values of FC between CYR61 and MMP28 ranged from 1.17 to 30.56 in the GC group across different datasets, while in the non-GC group the median values of FC ranged from 0.76 to 0.89 (Additional file 7: Table S4). Similar results for the gene pair of CYR61 and ACOX1 were also observed (Additional files 7 and 8). Notably, two genes with high expression levels in a sample can hardly reach large FC even if the absolute expression level difference between the two genes is rather large. Besides, two genes with low expression levels in a sample may reach large FC simply due to large measurement variations [45]. To more clearly show the quantitative expression level difference of two genes in each of the signature gene pairs, we also calculated the value of the expression level of CYR61 minus the expression level of MMP28 (ACOX1) in a sample as a measure to show the difference of the two genes consisting of the signature gene pairs (Additional files 9 and 10**).** The median values of the subtraction of MMP28 from CYR61 ranged from 1.30 to 1868.50 in the GC group across different datasets, while in the non-GC group the median values ranged from − 2.29 to − 0.73 (Additional file 9: Table S5). The results were similar for the gene pair of CYR61 and ACOX1 (Additional files 9 and 10). The subtraction values were quite different for different platforms. However, they varied even in the same platform. For example, the median values of the subtraction of MMP28 from CYR61 in GC group ranged from 2.84 to 1868.5 for GPL6947 (Additional files 9 and 10). The above results showed that the subtle quantitative difference (such as FC and subtraction) of each of the signature gene pairs is quite different across different samples for both the GC and non-GC groups because the quantitative gene expression measurements are affected by the measurement batch effects and many other factors such like the sample quality [29, 31, 46]. However, the REOs of the gene pairs in each group are very stable.

We additionally evaluated the performance of the signature on other types of cancers including liver, colorectal and pancreatic cancers (Additional file 11: Table S6). As shown in Additional file 12: Table S7, the results showed that the signature was unsuitable for these types of cancers. Notably, the signature can classify cancer tissues of liver, colorectum and pancreas as cancer although it cannot correctly classify most non-cancer tissues as non-cancer. The signature genes, including CYR61, MMP28 and ACOX1, may play important roles in the initiation and progression of cancer. As shown in Additional file 13: Table S8, CYR61 and MMP28 are involved in functions such as cell proliferation, differentiation or metastasis related to the initiation and progression of cancer. ACOX1 has been reported to regulate cancer development [47] and its dysfunction is linked to hepatocarcinogenesis [48] and migration and invasion of colorectal cancer cells [49]. Therefore, the stable REOs of genes in the signature may be an inherent feature of cancer which deserves our future study.

## Conclusions

In summary, we have developed a transcriptional qualitative signature for GC diagnosis, which exhibits robust and excellent performance in data measured by different laboratories with different platforms.

## Additional files

**Additional file 1: Table S1.** The baseline characteristics of seven GC patients.

**Additional file 2.** The code to identify the signature for GC diagnosis. All of the analysis programs to develop the diagnostic signature were written using the R language (R 3.1.3).

**Additional file 3: Table S2.** The number of stable and reversal gene pairs identified in the training data.

**Additional file 4.** The REOs of the top gene pairs. The distributions of REOs of the top six gene pairs in each of the training datasets.

**Additional file 5: Table S3.** The classification accuracy of the signature in each of the training datasets.

**Additional file 6.** The REOs of the signature gene pairs. The distributions of REOs of the signature gene pairs in each of the validation datasets.

**Additional file 7: Table S4.** The median values of FC of each signature gene pair across different datasets for the GC, non-GC and GC adjacent-normal groups.

**Additional file 8: Fig. S1.** The distributions of FCs of each signature gene pairs across different datasets for the GC, non-GC and GC adjacent-normal groups. Gene pair1 and gene pair2 represent gene pairs of CYR61-MMP28 and CYR61-ACOX1, respectively.

**Additional file 9: Table S5.** The median values of the subtraction of two gene expression levels across different datasets for the GC, non-GC and GC adjacent-normal groups.

**Additional file 10: Fig. S2.** The distributions of the subtraction of two gene expression levels across different datasets for the GC, non-GC, and GC adjacent-normal groups.

**Additional file 11: Table S6.** The datasets of cancer and non-cancer tissues for liver, colorectum and pancreas.

**Additional file 12: Table S7.** The performance of the signature in classifying cancer and non-cancer tissues of liver, colorectum and pancreas.

**Additional file 13: Table S8.** The summary of genes in the signature.

## Abbreviations

GC: gastric cancer
REOs: relative expression orderings
GEO: Gene Expression Omnibus
TCGA: The Cancer Genome Atlas
AUC: area under curve
ROC: receiver operating characteristic
CI: confidence intervals
FC: fold changes
