# Supplementary material for: A robust qualitative transcriptional signature for the correct pathological diagnosis of gastric cancer
Source: J Transl Med. 2019 Feb 28;17:63. doi: 10.1186/s12967-019-1816-4 (PMC6394047; doi:10.1186/s12967-019-1816-4)
Supplement: Supplementary file 1 — Additional file 1: Table S1. The baseline characteristics of seven GC patients. [file 12967_2019_1816_MOESM1_ESM.doc]

**Table S1.** The baseline characteristics of seven GC patients.

| Patient | Sex | Age | Tumor size | Subtype | pTNM | BMI |
| --- | --- | --- | --- | --- | --- | --- |
| GC 1 | female | 65 | 45mm | Diffuse | IIIC | 21.58 |
| GC 2 | female | 67 | 25mm | Intestinal | IIIB | 25.32 |
| GC 3 | male | 70 | 50mm | NA | IIA | 24.92 |
| GC 4 | male | 57 | 80mm | Diffuse | IIIA | 22.10 |
| GC 5 | female | 57 | 40mm | Diffuse | IIA | 17.94 |
| GC 6 | female | 67 | 75mm | Diffuse | IIIC | 24.00 |
| GC 7 | male | 58 | 40mm | Diffuse | IIIB | 26.30 |
