# Supplementary material for: A robust qualitative transcriptional signature for the correct pathological diagnosis of gastric cancer
Source: J Transl Med. 2019 Feb 28;17:63. doi: 10.1186/s12967-019-1816-4 (PMC6394047; doi:10.1186/s12967-019-1816-4)
Supplement: Supplementary file 2 — Additional file 2. The code to identify the signature for GC diagnosis. All of the analysis programs to develop the diagnostic signature were written using the R language (R 3.1.3). [file 12967_2019_1816_MOESM2_ESM.pdf]

The code to identify the signature for GC diagnosis.

All of the analysis programs to develop the diagnostic signature were achieved using the R language.

Step 1. Identify stable gene pairs

```
slec_stable_pair<-function(expE,cutoff) {
gid<-expE[,1]
exp<-expE[,-1]
len=length(gid)-1
m=length(exp[1,])
pair=matrix(nrow=choose(length(gid),2),ncol=3)
j=1
for (i in 1:len)
{
	len-i
	RE=exp[i*ones(len-i+1,1),]-exp[i+1:end,]>0
	ratio=rowsum(RE)/m
	pair[j:j+length[RE[,1]]-1,]=c(gid[i]*ones[len-i+1,1],gid[i+1:end,],ratio)
	j=j+length[RE[,1]]
}
stable_pair_1=pair[pair[,3]>=cutoff,1:2]
stable_pair_2=pair[pair[,3]<=1-cutoff,2:1]
stable_pair=rbind(stable_pair_1,stable_pair_2)
return(stable_pair)
}
```

# expE: the gene expression profiles of GC or non-GC
# cutoff: 99% or other values defined by users.

Step 2. Identify reversal gene pairs

```
Trainingcancerexp_stable_pair=slec_stable_pair(trainingcancerexp,0.99);
```

#Stable pairs of GC
#trainingcancerexp represents the gene expression profiles of GC in training data

```
Trainingnormalexp_stable_pair=slec_stable_pair(trainingnormalexp,0.99);
```

#Stable pairs of normal gastric tissues
#trainingnormalexp represents the gene expression profiles of normal gastric tissues in training data

```
trainingyanexp_stable_pair=slec_stable_pair(trainingyanexp,0.99);
```

#Stable pairs of gastritis tissues
#trainingyanexp represents the gene expression profiles of gastritis tissues in training data

```
a<-trainingyanexp_stable_pair
b<-trainingnormalexp_stable_pair
res <- matrix(0,1,dim(b)[2])
i <-2
for(i in 1:dim(a)[1]){
	temp <- rep(a[i,],dim(b)[1])
	temp <- matrix(temp,dim(b)[1],dim(b)[2],byrow=T)
	c <- temp==b
	apply(c,1,all)
		index <- apply(c,1,all)
	res <- rbind(res,b[index,])
}
normal_yan_commonpairs<-res[2:dim(res)[1],]
#The same stable gene pairs between normal gastric tissues and gastritis tissues

trainingcancerexp_stable_pair<-trainingcancerexp_stable_pair[,2:1]
a<-trainingcancerexp_stable_pair
b<-normal_yan_commonpairs

res <- matrix(0,1,dim(b)[2])
i <-2
for(i in 1:dim(a)[1]){
	temp <- rep(a[i,],dim(b)[1])
	temp <- matrix(temp,dim(b)[1],dim(b)[2],byrow=T)
	c <- temp==b
	apply(c,1,all)
		index <- apply(c,1,all)
	res <- rbind(res,b[index,])
}
reverse_pairs<-res[2:dim(res)[1],]
#The reversal gene pairs between GC and non-GC tissues
```

Step 3. Identify the top gene pairs

```
training_cancer_exp
training_normal_yan_exp=cbind(trainingyanexp,trainingnormalexp)
reverse_pairs
rank_def_matrix_cancer=[];
for (i in 1:length(reverse_pairs[,1])){
i
gene_1=reverse_pairs[i,1]
gene_2=reverse_pairs[i,2]
gene_1_index=which(geneid==gene_1)
```

```
gene_2_index=which(geneid==gene_2)
for (j in 1:length(training_cancer_exp[1,])){
j_sample_exp=training_cancer_exp[,j]
index=order(j_sample_exp,decreasing=TRUE)
gene_1_rank=which(index==gene_1_index)
gene_2_rank=which(index==gene_2_index)
rank_def=abs(gene_1_rank-gene_2_rank)
rank_def_matrix_cancer[i,j]=rank_def
}
}

mean_rank_def_cancer=apply(rank_def_matrix_cancer,1,mean)
rank_def_matrix_normalyan=[];
for( i in 1:length(reverse_pairs(:,1))){
i
gene_1=reverse_pairs[i,1]
gene_2=reverse_pairs[i,2]
gene_1_index=which(geneid==gene_1)
gene_2_index=which(geneid==gene_2)
for (j in 1:length(training_normal_yan_exp[1,])){
j_sample_exp=training_normal_yan_exp[,j]
index=order(j_sample_exp,decreasing=TRUE)
gene_1_rank=which(index==gene_1_index)
gene_2_rank=which(index==gene_2_index)
rank_def=abs(gene_1_rank-gene_2_rank)
rank_def_matrix_normalyan[i,j]=rank_def;
}
}
mean_rank_def_normalyan=apply(rank_def_matrix_normalyan,1,mean)
average_rank_def=sqrt(mean_rank_def_cancer*mean_rank_def_normalyan);
top_index=order(average_rank_def,decreasing=TRUE)
top1_index=top_index[1,]
top1_gene_pairs=reverse_pairs(top1_index,:);
```

Step 4. The rule to identify GC and non-GC tissues

```
C<-cancer_exp
B<-canceradjacent_exp

top2_index11=which(geneid==top2_gene_pairs[1,1])
top2_index12=which(geneid==top2_gene_pairs[1,2])
top2_index21=which(geneid==top2_gene_pairs[2,1])
top2_index22=which(geneid==top2_gene_pairs[2,2])
```

```
top2_gene11_exp_cancer_canceradjacent=C[top2_index11,]    #B[top2_index11,]
top2_gene12_exp_cancer_canceradjacent=C[top2_index12,]    #B[top2_index12,]
top2_gene21_exp_cancer_canceradjacent=C[top2_index21,]    #B[top2_index21,]
top2_gene22_exp_cancer_canceradjacent=C[top2_index22,]    #B[top2_index22,]

top2_pair1_def_exp_cancer_canceradjacent<-
top2_gene11_exp_cancer_canceradjacent-top2_gene12_exp_cancer_canceradjacent
top2_pair2_def_exp_cancer_canceradjacent<-
top2_gene21_exp_cancer_canceradjacent-top2_gene22_exp_cancer_canceradjacent

top2_def_exp_cancer_canceradjacent<-
rbind(top2_pair1_def_exp_cancer_canceradjacent,top2_pair2_def_exp_cancer_cancer
adjacent)
num<-matrix(,nrow=1,ncol=length(top2_def_exp_cancer_canceradjacent[1,]))
for (i in 1:length(top2_def_exp_cancer_canceradjacent[1,])){
num[1,i]=length(which(top2_def_exp_cancer_canceradjacent[,i]>0))
}
len<-2
th<-len/2
top2_half=length(which(num>=th))
```
