# Supplementary material for: A robust qualitative transcriptional signature for the correct pathological diagnosis of gastric cancer
Source: J Transl Med. 2019 Feb 28;17:63. doi: 10.1186/s12967-019-1816-4 (PMC6394047; doi:10.1186/s12967-019-1816-4)
Supplement: Supplementary file 3 — Additional file 3: Table S2. The number of stable and reversal gene pairs identified in the training data. [file 12967_2019_1816_MOESM3_ESM.doc]

**Table S2.** The number of stable and reversal gene pairs identified in the training data.

| Sample type | Number of stable gene pairs | Number of overlap stable gene pairs | Number of reversal gene pairs |
| --- | --- | --- | --- |
| Normal | 46640259 | 32483417 | 6 |
| Gastritis | 36229525 |
| Gastric cancer | 18932505 | - |
