# Supplementary material for: A robust qualitative transcriptional signature for the correct pathological diagnosis of gastric cancer
Source: J Transl Med. 2019 Feb 28;17:63. doi: 10.1186/s12967-019-1816-4 (PMC6394047; doi:10.1186/s12967-019-1816-4)
Supplement: Supplementary file 5 — Additional file 5: Table S3. The classification accuracy of the signature in each of the training datasets. [file 12967_2019_1816_MOESM5_ESM.doc]

**Table S3.** The classification accuracy of the signature in each of the training datasets.

| Platforms | Dataset | Number (Sensitivity) of GC tissues | Number (specificity) of non-GC tissues |
| --- | --- | --- | --- |
| Affymetrix | GSE54129 | 111(100.00%) | 21(100.00%) |
|  | GSE54043 | - | 10(100.00%) |
|  | GSE42252 | 5 (100.00%) | - |
|  | GSE38749 | 15 (100.00%) | - |
|  | GSE51725 | 8(100.00%) | - |
|  | GSE79973 | 10(100.00%) | - |
|  | GSE57303 | 70(100.00%) | - |
|  | GSE13911 | 38(100.00%) | - |
| Illumina | GSE28541 | 40(100.00%) | - |
|  | GSE29998 | 50(100.00%) | - |
|  | GSE27411 | - | 18(100.00%) |
