# Supplementary material for: A robust qualitative transcriptional signature for the correct pathological diagnosis of gastric cancer
Source: J Transl Med. 2019 Feb 28;17:63. doi: 10.1186/s12967-019-1816-4 (PMC6394047; doi:10.1186/s12967-019-1816-4)
Supplement: Supplementary file 7 — Additional file 7: Table S4. The median values of FC of each signature gene pair across different datasets for the GC, non-GC and GC adjacent-normal groups. [file 12967_2019_1816_MOESM7_ESM.doc]

**Table S4.** The median values of FC of each signature gene pair across different datasets for the GC, non-GC and GC adjacent-normal groups.

| Dataset | GC | | Non-GC | | Adjacent-normal | |
| --- | --- | --- | --- | --- | --- | --- |
|  | Gene pair1 | Gene pair2 | Gene pair1 | Gene pair2 | Gene pair1 | Gene pair2 |
| GSE42252 | 1.45 | 1.50 | - | - | - | - |
| GSE38749 | 1.35 | 1.49 | - | - | - | - |
| GSE51725 | 1.74 | 1.48 | - | - | - | - |
| GSE79973 | 1.56 | 1.50 | - | - | - | - |
| GSE57303 | 1.89 | 1.64 | - | - | - | - |
| GSE13911 | 1.44 | 1.37 | - | - | - | - |
| GSE28541 | 1.19 | 1.13 | - | - | - | - |
| GSE29998 | 30.56 | 40.56 | - | - | - | - |
| GSE52138 | 1.62 | 1.18 | - | - | 1.27 | 0.98 |
| GSE14210 | 1.62 | 1.18 | - | - | - | - |
| GSE29272 | 1.85 | 1.58 | - | - | 1.74 | 1.50 |
| GSE34942 | 1.70 | 1.60 | - | - | - | - |
| GSE22377 | 1.59 | 1.49 | - | - | - | - |
| GSE13861 | 1.45 | 1.46 | - | - | 1.55 | 1.56 |
| GSE38024 | 1.26 | 1.28 | - | - | - | - |
| GSE26899 | 1.40 | 1.49 | - | - | 1.36 | 1.46 |
| GSE19826 | 1.86 | 1.70 | - | - | 1.73 | 1.58 |
| GSE51105 | 1.78 | 1.58 | - | - | - | - |
| GSE35809 | 1.78 | 1.58 | - | - | - | - |
| GSE26253 | 1.17 | 1.36 | - | - | - | - |
| GSE15459 | 1.53 | 1.57 | - | - | - | - |
| GSE62254 | 1.40 | 1.58 | - | - | - | - |
| GSE84437 | 4.42 | 5.73 | - | - | - | - |
| GSE26942 | 1.46 | 1.36 | - | - | 1.45 | 1.36 |
| TCGA | 9.02 | 3.02 | - | - | - | - |
| GSE54129 | 1.64 | 1.66 | 0.76 | 0.77 | - | - |
| GSE27411 | - | - | 0.80 | 0.76 | - | - |
| GSE54043 | - | - | 0.79 | 0.78 | - | - |
| GSE5081 | - | - | 0.83 | 0.84 | - | - |
| GSE106656 | - | - | 0.86 | 0.69 | - | - |
| GSE60662 | - | - | 0.76 | 0.61 | - | - |
| GSE34619 | - | - | 0.89 | 0.67 | - | - |

Gene pair1 and gene pair2 represent CYR61-MMP28 and CYR61-ACOX1, respectively.
