# Supplementary figures and images for: A robust qualitative transcriptional signature for the correct pathological diagnosis of gastric cancer
Source: J Transl Med. 2019 Feb 28;17:63. doi: 10.1186/s12967-019-1816-4 (PMC6394047; doi:10.1186/s12967-019-1816-4)

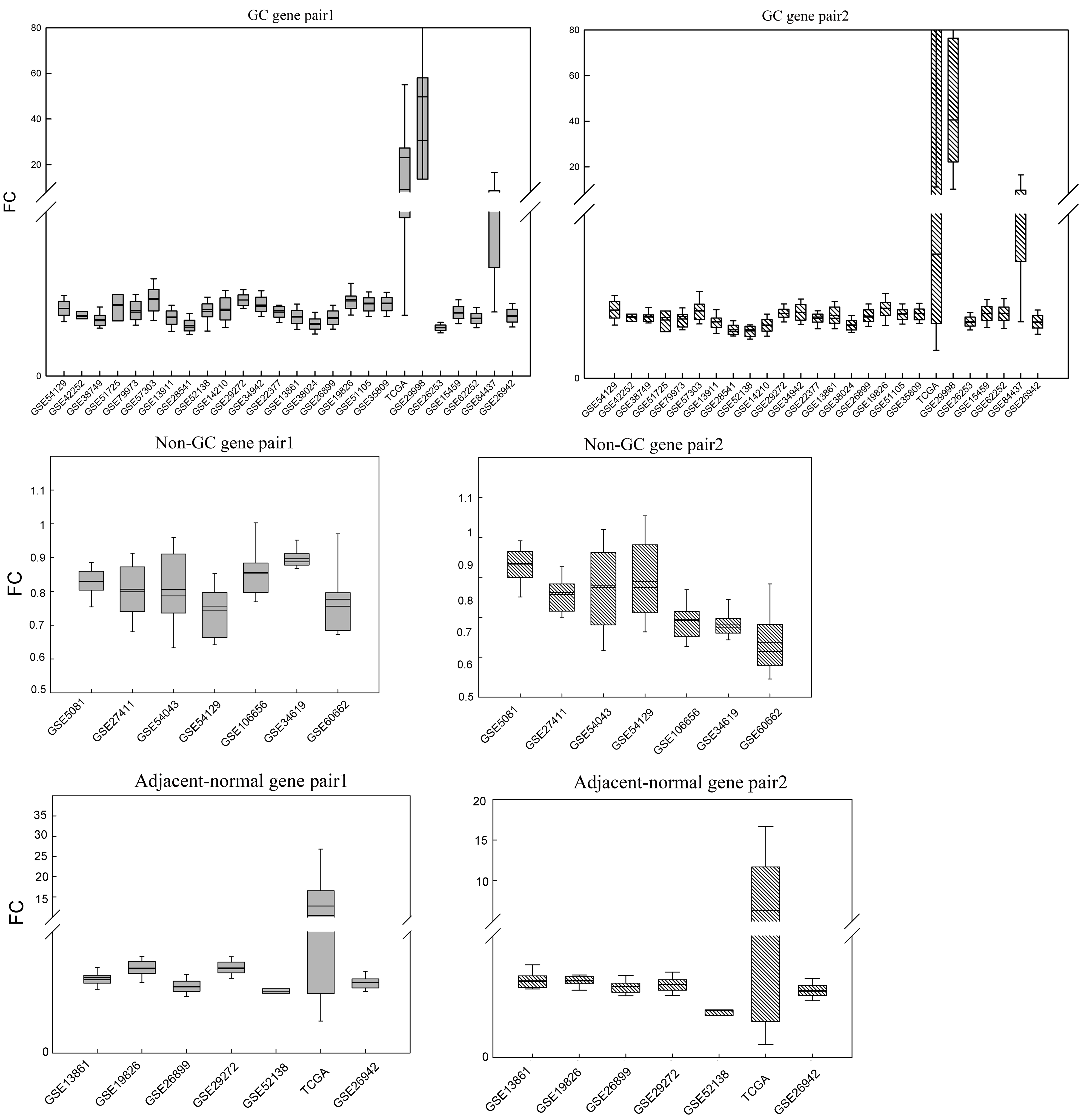

Supplement: Supplementary file 8 — Additional file 8: Fig. S1. The distributions of FCs of each signature gene pairs across different datasets for the GC, non-GC and GC adjacent-normal groups. Gene pair1 and gene pair2 represent gene pairs of CYR61-MMP28 and CYR61-ACOX1, respectively. [file 12967_2019_1816_MOESM8_ESM.tif]

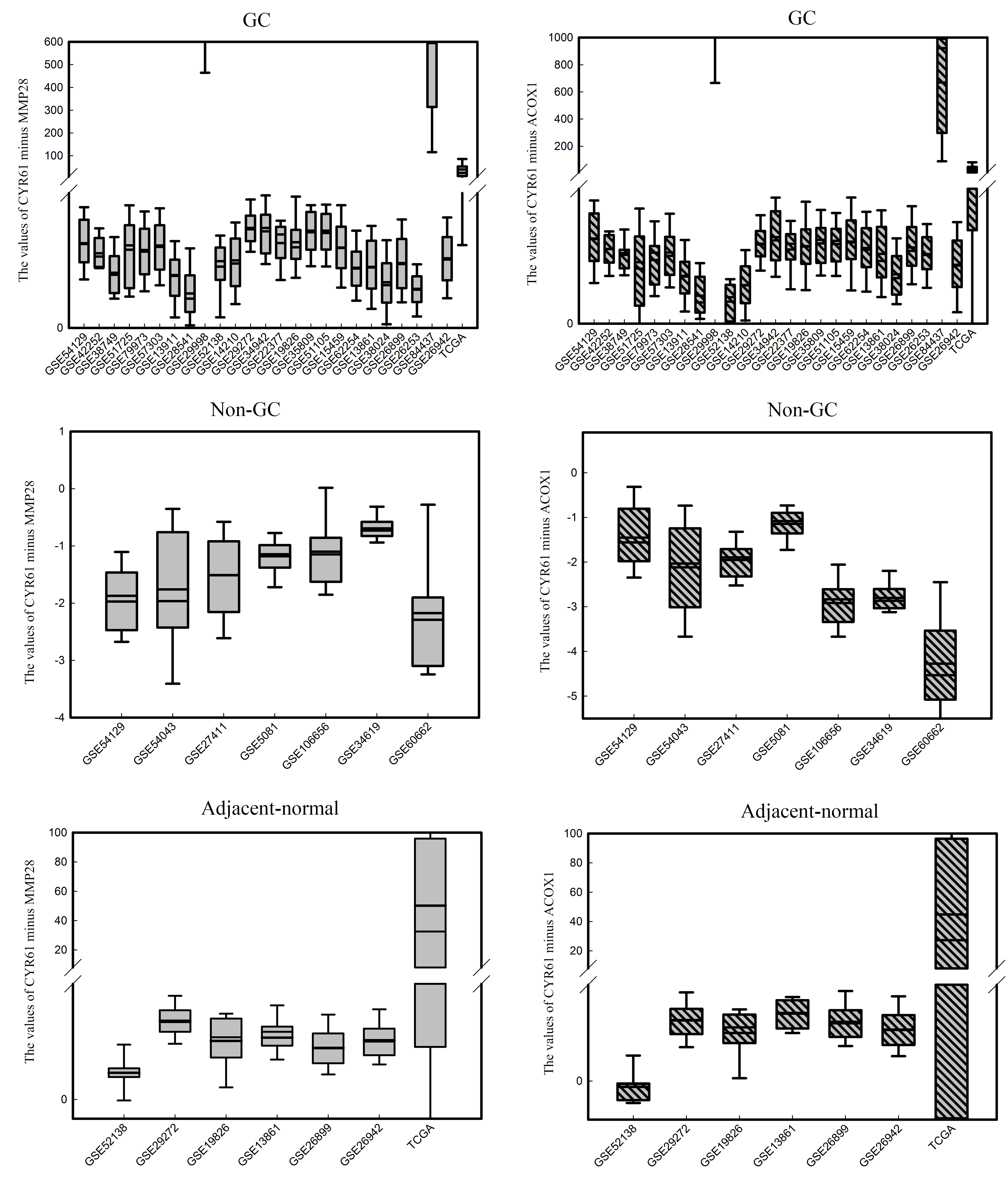

Supplement: Supplementary file 10 — Additional file 10: Fig. S2. The distributions of the subtraction of two gene expression levels across different datasets for the GC, non-GC, and GC adjacent-normal groups. [file 12967_2019_1816_MOESM10_ESM.tif]
