# Supplementary material for: A robust qualitative transcriptional signature for the correct pathological diagnosis of gastric cancer
Source: J Transl Med. 2019 Feb 28;17:63. doi: 10.1186/s12967-019-1816-4 (PMC6394047; doi:10.1186/s12967-019-1816-4)
Supplement: Supplementary file 9 — Additional file 9: Table S5. The median values of the subtraction of two gene expression levels across different datasets for the GC, non-GC and GC adjacent-normal groups. [file 12967_2019_1816_MOESM9_ESM.doc]

**Table S5.** The median values of the subtraction of two gene expression levels across different datasets for the GC, non-GC and GC adjacent-normal groups.

| Dataset | Platform | GC | | Non-GC | | Adjacent-normal | |
| --- | --- | --- | --- | --- | --- | --- | --- |
|  |  | Gene pair1 | Gene pair2 | Gene pair1 | Gene pair2 | Gene pair1 | Gene pair2 |
| GSE54129 | GPL570 | 3.77 | 3.76 | -1.97 | -1.56 | - | - |
| GSE42252 | GPL570 | 3.18 | 3.31 | - | - | - | - |
| GSE38749 | GPL570 | 2.36 | 3.13 | - | - | - | - |
| GSE51725 | GPL570 | 3.67 | 2.73 | - | - | - | - |
| GSE79973 | GPL570 | 3.47 | 3.16 | - | - | - | - |
| GSE57303 | GPL570 | 3.61 | 3.01 | - | - | - | - |
| GSE13911 | GPL570 | 2.35 | 2.09 | - | - | - | - |
| GSE28541 | GPL13376 | 1.30 | 0.94 | - | - | - | - |
| GSE29998 | GPL6947 | 1868.50 | 1953.50 | - | - | - | - |
| GSE52138 | GPL96 | 2.97 | 1.18 | - | - | 1.37 | -0.15 |
| GSE14210 | GPL571 | 3.00 | 1.70 | - | - | - | - |
| GSE29272 | GPL96 | 4.39 | 3.52 | - | - | 4.01 | 3.17 |
| GSE34942 | GPL570 | 4.45 | 3.70 | - | - | - | - |
| GSE22377 | GPL570 | 4.16 | 3.55 | - | - | - | - |
| GSE13861 | GPL6884 | 2.69 | 2.78 | - | - | 3.51 | 3.53 |
| GSE38024 | GPL10558 | 1.90 | 2.02 | - | - | - | - |
| GSE26899 | GPL6947 | 2.84 | 3.23 | - | - | 2.65 | 3.00 |
| GSE19826 | GPL570 | 3.59 | 3.43 | - | - | 3.23 | 2.78 |
| GSE51105 | GPL570 | 4.32 | 3.68 | - | - | - | - |
| GSE35809 | GPL570 | 4.33 | 3.73 | - | - | - | - |
| GSE26253 | [GPL8432](https://www.ncbi.nlm.nih.gov/geo/query/acc.cgi?acc=GPL8432) | 1.74 | 3.10 | - | - | - | - |
| GSE15459 | [GPL570](https://www.ncbi.nlm.nih.gov/geo/query/acc.cgi?acc=GPL570) | 3.55 | 3.61 | - | - | - | - |
| GSE62254 | [GPL570](https://www.ncbi.nlm.nih.gov/geo/query/acc.cgi?acc=GPL570) | 2.64 | 3.63 | - | - | - | - |
| GSE84437 | [GPL6947](https://www.ncbi.nlm.nih.gov/geo/query/acc.cgi?acc=GPL6947) | 633.06 | 668.47 | - | - | - | - |
| GSE26942 | [GPL6947](https://www.ncbi.nlm.nih.gov/geo/query/acc.cgi?acc=GPL6947) | 3.04 | 2.62 | - | - | 3.00 | 2.65 |
| TCGA | RNA-seq | 24.90 | 18.92 | - | - | - | - |
| GSE5081 | GPL570 | - | - | -1.17 | -1.04 | - | - |
| GSE27411 | GPL6255 | - | - | -1.51 | -1.89 | - | - |
| GSE54043 | GPL570 | - | - | -1.18 | -1.64 | - | - |
| GSE106656 | GPL6244 | - | - | -1.10 | -2.84 | - | - |
| GSE60662 | GPL13497 | - | - | -2.29 | -4.53 | - | - |
| GSE34619 | GPL6244 | - | - | -0.73 | -2.87 | - | - |

Gene pair1 and gene pair2 represent CYR61-MMP28 and CYR61-ACOX1, respectively.
