# Supplementary material for: A robust qualitative transcriptional signature for the correct pathological diagnosis of gastric cancer
Source: J Transl Med. 2019 Feb 28;17:63. doi: 10.1186/s12967-019-1816-4 (PMC6394047; doi:10.1186/s12967-019-1816-4)
Supplement: Supplementary file 11 — Additional file 11: Table S6. The datasets of cancer and non-cancer tissues for liver, colorectum and pancreas. [file 12967_2019_1816_MOESM11_ESM.doc]

**Table S5.** The datasets of cancer and non-cancer tissues for liver, colorectum and [pancreas](javascript:;).

| Dataset | Number of cancer tissues | Number of non-cancer tissues |
| --- | --- | --- |
| Liver |  |  |
| GSE17548 | 17 | - |
| GSE41804 | 20 | - |
| GSE62232 | 81 | - |
| GSE6764 | 35 | - |
| GSE39791 | 72 | - |
| GSE36376 | 193 | - |
| GSE63898 | 228 | - |
| GSE14323 | 37 | 60 |
| GSE17967 | - | 47 |
| GSE15654 | - | 151 |
| Colorectum |  |  |
| GSE12251 | - | 23 |
| GSE13367 | - | 16 |
| GSE14580 | - | 24 |
| GSE16879 | - | 43 |
| GSE36807 | - | 28 |
| GSE47908 | - | 39 |
| GSE9348 | 70 | - |
| GSE35452 | 46 | - |
| GSE45404 | 42 | - |
| GSE17536 | 177 | - |
| GSE18105 | 77 | - |
| GSE21510 | 104 | - |
| GSE23878 | 35 | - |
| GSE27854 | 115 | - |
| [Pancreas](javascript:;) |  |  |
| GSE91035 | 25 | 10 |
| GSE62452 | 69 | - |
| GSE56560 | 28 | 3 |
| GSE28735 | 45 | - |
| GSE22780 | 8 | - |
| GSE62165 | 118 | - |
| GSE60646 | 10 | - |
| GSE15471 | 39 | - |
| GSE71989 | 13 | 9 |
| E-MTAB-1791 | 195 | 115 |
| E-MEXP-804 | - | 9 |

The non-cancer tissues for liver are cirrhosis tissues of non-hepatocellular carcinoma patients. For colorectum, non-cancer tissues include normal and inflammatory bowel disease tissues. For [pancreas](javascript:;), non-cancer tissues include normal and [pancreatitis](javascript:;) tissues.
