# Supplementary material for: A robust qualitative transcriptional signature for the correct pathological diagnosis of gastric cancer
Source: J Transl Med. 2019 Feb 28;17:63. doi: 10.1186/s12967-019-1816-4 (PMC6394047; doi:10.1186/s12967-019-1816-4)
Supplement: Supplementary file 12 — Additional file 12: Table S7. The performance of the signature in classifying cancer and non-cancer tissues of liver, colorectum and pancreas. [file 12967_2019_1816_MOESM12_ESM.doc]

**Table S6.** The performance of the signature in classifying cancer and non-cancer tissues of liver, colorectum and pancreas.

| Dataset | Number (Sensitivity) of cancer tissues | Number (specificity) of non-cancer tissues |
| --- | --- | --- |
| Liver | 683 (97.95%) | 258 (15.12%) |
| colorectum | 904 (96.79%) | 206 (27.67%) |
| pancreas | 550 (99.64%) | 146 (0.00%) |
