# Supplementary material for: A robust qualitative transcriptional signature for the correct pathological diagnosis of gastric cancer
Source: J Transl Med. 2019 Feb 28;17:63. doi: 10.1186/s12967-019-1816-4 (PMC6394047; doi:10.1186/s12967-019-1816-4)
Supplement: Supplementary file 13 — Additional file 13: Table S8. The summary of genes in the signature. [file 12967_2019_1816_MOESM13_ESM.doc]

**Table S7.** The summary of genes in the signature.

| Gene name | Summary |
| --- | --- |
| CYR61 | The secreted protein encoded by this gene is growth factor-inducible and promotes the adhesion of endothelial cells. The encoded protein interacts with several integrins and with heparan sulfate proteoglycan. This protein also plays a role in cell proliferation, differentiation, angiogenesis, apoptosis, and extracellular matrix formation. |
| MMP28 | Proteins of the matrix metalloproteinase family are involved in the breakdown of extracellular matrix for both normal physiological processes, such as embryonic development, reproduction and tissue remodeling, and disease processes, such as asthma and metastasis. This gene encodes a secreted enzyme that degrades casein. Its expression pattern suggests that it plays a role in tissue homeostasis and in wound repair. Alternative splicing of this gene results in multiple transcript variants. |
| ACOX1 | The protein encoded by this gene is the first enzyme of the fatty acid beta-oxidation pathway, which catalyzes the desaturation of acyl-CoAs to 2-trans-enoyl-CoAs. It donates electrons directly to molecular oxygen, thereby producing hydrogen peroxide. Defects in this gene result in pseudoneonatal adrenoleukodystrophy, a disease that is characterized by accumulation of very long chain fatty acids. Alternatively spliced transcript variants encoding different isoforms have been identified. |
